# The Comparison of 940nm and 810nm Diode Laser Effects on the Repair of Inferior Alveolar Sensory Nerve Injury: A Clinical Trial

**DOI:** 10.30476/dentjods.2023.97393.2013

**Published:** 2024-09-01

**Authors:** Hooman Ebrahimi, Masoumeh Kargar, Reyhaneh Shoorgashti

**Affiliations:** 1 Dept. of Oral Medicine, Dental Faculty, Azad University of Medical Sciences, Tehran, Iran; 2 Dentist, Tehran, Iran

**Keywords:** Semiconductor Diode Laser, GaAlAs lasers, GaInP lasers, Mandibular nerve injuries, Inferior alveolar nerves, Low-level light therapy, Nerve regeneration, Photobiomodulation therapy

## Abstract

**Statement of the Problem:**

Healing of the inferior alveolar nerve injury during dental procedures is one of the biggest concerns of dentists. There are still debates on different treatment modalities.

**Purpose:**

This study aimed to compare the effect of 940nm and 810nm diode lasers on the repair of the inferior alveolar sensory nerve.

**Materials and Method:**

In this single-blinded randomized clinical trial, 39 patients with inferior alveolar nerve injury were divided into three groups: 1. 810nm laser irradiated, 2. 940nm laser irradiated, and 3. No laser irradiation (control group). All patients were treated in 12 sessions (3 days per week) and evaluated using a complete clinical neurosensory test (CNT), including brushstroke, 2-point discrimination, pinprick nociception, and thermal discrimination before and after treatment.

**Results:**

The mean dysesthesia of the patient treated with 810nm diode laser was significantly lower than the control group in all sessions (the 1^st^ (*p*= 0.003), 3^rd^ (*p*= 0.008), 7^th^ (*p*= 0.006), and 12^th^ sessions (*p*= 0.005)). The 810nm laser resulted in more satisfaction in patients than the control group in almost all sessions (1^st^ (*p*< 0.001), 7^th^ (*p*= 0.028), and 12^th^ (*p*= 0.006)). More patient satisfaction was seen in the 1^st^ and 3^rd^ sessions in the 810nm laser than in the 980nm laser (*p*< 0.001 and *p*= 0.003, respectively).

**Conclusion:**

810nm diode laser can be better than 940nm in repairing inferior alveolar sensory nerve damage.

## Introduction

Inferior alveolar nerve injury during dental procedures is widespread and one of the biggest concerns among dentists [ 1 - 3 ]. The inferior alveolar nerve and the lingual nerve can be injured during local anesthesia injection, root canal therapy, implantation, dentoalveolar surgeries, and particularly third molar extraction [ 4 - 7 ]. The prevalence of inferior alveolar nerve damage can be 20 to 40% [ 8 - 9 ]. Some factors that can increase the risk of this damage are surgery complications, lack of enough experience, and a short distance between the surgical area and the inferior alveolar nerve [ 10 ]. Inferior alveolar nerve injury can result in continuous pain, dysarthria, dysphasia, or even disability in common activities like kissing or makeup. This injury can be temporary or permanent and represents itself with anesthesia, paresthesia, or dysesthesia [ 1 , 5 ]. 

Immediate intervention in inferior alveolar nerve injury is challenging due to the difficulty in estimating the amount of damage and access to the nerve surrounded by alveolar bones [ 4 ]. In these cases, the first common treatment has been grafting, in which the damaged part is replaced, and the second one is to put two separate parts of the nerves near one another to use the growth potential of the peripheral axons for healing [ 11 - 12 ]. Vascular grafts can also be used, but the success rate of this approach is poor because of the lack of enough strength in vessels and vascular collapse [ 11 ].

Photobiomodulation (PBM) is a non-aggressive procedure that can be used in different injuries and conditions. There are various positive reports regarding applying PBM for diseases and damages related to either the central or peripheral nervous system [ 7 , 13 ]. It is established that PBM can stimulate axon growth and nerve reconstruction in the spinal cord and peripheral nerves [ 14 - 15 ]. Since there has not been any definite treatment for nerve injuries and because of advancements in technology, using lasers for nerve-injured cases has arisen. Low-level laser therapy (LLLT) has recently been introduced as a treatment with a good prognosis for inferior alveolar nerve injuries [ 16 - 17 ].

However, only some studies are available regarding applying this method, and the results of conducted studies are controversial [ 17 - 20 ]. Therefore, this study was conducted to compare the effectiveness of two lasers, 810nm and 940nm, on repairing inferior alveolar nerve injuries in patients of the Laser Research Center of Dentistry, Tehran University of Medical Science, Tehran, Iran.

## Materials and Method

In the present study, the ethical committee registered and approved the methodologies (ethical code= IR.IAU. DENTAL.REC.1398.008) and it was registered in the Iranian Registry of Clinical Trials under the registration number IRCT 20190520043644N1.

### Sample size calculation

The sample size of this study was calculated as 13 in each group (total sample size=39), based on a previous study [ 21 ] considering α= 0.05 and β=0.2, using the Advanced Repeated Measures ANOVA Power Analysis option in PASS II. 

### Eligibility criteria

### Inclusion criteria

Men and women aged between 18 and 65 and at most, six months passed from their inferior alveolar sensory nerve injury due to dental procedures were included in this study. They have not received any treatment and must not have taken any medications that might affect either central or peripheral nervous systems during the past month.

### Exclusion criteria

Patients, who were pregnant, had systemic diseases, addiction experience, or smoking habits were excluded from the study.

### Treatment Procedure

At first, the treatment procedure was explained to all patients, and informed consent was signed by the participants. All patients were prescribed 300 milligrams (mg) of B1 vitamin, one tablet each morning, and 100mg of Gabapentin, one capsule each noon. They were randomly divided into three groups using permuted block randomization. In group 1, participants were irradiated using an 810nm *GaAlAs* laser (810 nm gallium aluminum arsenide semiconductor diode laser, Thor Company, England). In group 2, participants were irradiated using a 940nm *GaInP* laser (940nm gallium indium phosphide laser, Biolase Company, USA); and in group 3 (control group), no laser irradiation was applied.

For blindness, laser irradiation and a complete clinical neurosensory test (CNT) were done by two operators. In group1 and 2, lasers were irradiated to 3 areas: 1. Inferior alveolar nerve entrance to the mandibular foramen, 2. Lip, and 3. A space between the two mentioned areas with equal distance from both. Patients were treated in 12 sessions (3 days per week), and in each session, lasers were irradiated with 200mW power for 20 seconds on all three areas. Irradiation dose was $\mathrm{5.09}\frac{j}{{\mathrm{cm}}^{2}}$ calculated based on the spot area, which was 1cm^2^ and using $E_{D}=\frac{\mathrm{P\times T}}{A}$ formula:

$E_{D}=\frac{\mathrm{P\times T}}{A}=\frac{0.2\times 20}{0.786}=\mathrm{5.09}\frac{j}{{\mathrm{cm}}^{2}}$

Dysesthesia evaluating tests and CNT before and after treatment were used to assess the sensory and neuropathic disorders. CNT included four objective and one subjective test, including brushstroke and 2-point discrimination (level A tests) and thermal discrimination and pinprick nociception (level B tests) [ 21 ]. In the brushstroke test, patients’ reactions were recorded using a micro brush in vertical and horizontal movements on their skin. Patients who were able to detect either vertical or horizontal movements were categorized into one group, and those who could detect both movement directions were classified into another group (Table 1) [ 22 ]. In a 2-point discrimination test, >10mm distances were recorded. In this test, the physician randomly touched two points on the patient’s skin and asked whether they felt one or two touched areas.

**Table 1:** The frequency and percentage of brushstroke detection

Evaluation session	Groups	Non-detected N. (%)	Detection of horizontal OR vertical N. (%)	Detection of horizontal AND vertical N. (%)	Total N. (%)	*p* Value
Before treatment	Control	-	-	-	-	0.88
	810 nm laser	5 (38.5)	8 (61.5)	0 (0.0)	13 (100)	
	940 nm laser	5 (38.5)	5 (38.5)	3 (23.1)	13 (100)	
	Total	10 (38.5)	13 (50)	3 (11.5)	26 (100)	
1^st^ session	Control	5 (38.5)	5 (38.5)	3 (23.1)	13 (100)	0.592
	810 nm laser	2 (15.4)	9 (69.2)	2 (15.4)	13 (100)	
	940 nm laser	3 (23.1)	7 (53.8)	3 (23.1)	13 (100)	
	Total	10 (25.6)	21 (53.9)	8 (20.5)	39 (100)	
3^rd^ session	Control	4 (30.8)	6 (46.2)	3 (23.1)	13 (100)	0.767
	810 nm laser	2 (15.4)	9 (69.2)	2 (15.4)	13 (100)	
	940 nm laser	2 (15.4)	8 (61.5)	3 (23.1)	13 (100)	
	Total	8 (20.5)	23 (59)	8 (20.5)	39 (100)	
7^th^ session	Control	3 (23.1)	6 (46.2)	4 (30.8)	13 (100)	0.241
	810 nm laser	0 (0.0)	7 (53.8)	6 (46.2)	13 (100)	
	940 nm laser	1 (7.7)	5 (38.5)	7 (53.8)	13 (100)	
	Total	4 (10.3)	18 (46.2)	17 (43.6)	39 (100)	
12^th^ session	Control	1 (7.7)	5 (38.5)	7 (53.8)	13 (100)	0.628
	810 nm laser	0 (0.0)	4 (30.8)	9 (69.2)	13 (100)	
	940 nm laser	0 (0.0)	5 (38.5)	8 (61.5)	13 (100)	
	Total	1 (2.6)	14 (35.9)	24 (61.5)	39 (100)	

In this table shows the frequency and percentage of brushstroke test detection. The frequency of the detection of this test was the variant between the 3 groups. After 12 treatment sessions, most of the patients were able to detect brush strokes.

This examination continued until the least distance in which patients felt two areas [ 23 ]. In the pinprick nociception test, the patients’ skins were tapped frequently using a dental explorer, and they were asked about the number of taps. If patients detected less than 30% of all taps, it would be considered a negative response [ 21 ]. In thermal discrimination, the thermal test was applied using a thermode (thermal probe) on the skin. The thermal thresholds of patients were recorded when they reacted to the thermal changes. Patients who responded to either heat or cold stimuli were categorized in the “detection of heat OR cold” group, and others who were able to detect both were classified as “detection of heat AND cold” (Table 2) [ 22 ].

**Table 2:** The frequency and percentage of thermal test detection

Evaluation session	Groups	Non-detected N. (%)	Detection of heat OR cold N. (%)	Detection of heat AND cold N. (%)	Total N. (%)	*p* Value
Before treatment	Control	2 (15.4)	6 (46.2)	5 (38.5)	13 (100)	0.200
	810 nm laser	6 (46.2)	5 (38.5)	2 (15.4)	13 (100)	
	940 nm laser	5 (38.5)	5 (38.5)	3 (23.1)	13 (100)	
	Total	13 (33.3)	16 (41)	10 (25.6)	39 (100)	
1^st^ session	Control	2 (15.4)	5 (38.5)	6 (46.2)	13 (100)	0.564
	810 nm laser	4 (30.7)	6 (46.2)	3 (23.1)	13 (100)	
	940 nm laser	4 (30.7)	4 (30.7)	5 (38.5)	13 (100)	
	Total	10 (25.6)	15 (38.5)	14 (35.9)	39 (100)	
3^rd^ session	Control	1 (7.7)	6 (46.2)	6 (46.2)	13 (100)	0.230
	810 nm laser	2 (15.4)	8 (61.5)	3 (23.1)	13 (100)	
	940 nm laser	0 (0.0)	7 (53.8)	6 (46.2)	13 (100)	
	Total	3 (7.7)	21 (53.8)	15 (38.5)	39 (100)	
7^th^ session	Control	1 (7.7)	4 (30.8)	8 (61.5)	13 (100)	0.230
	810 nm laser	2 (15.4)	5 (38.5)	6 (46.2)	13 (100)	
	940 nm laser	0 (0.0)	4 (30.8)	9 (69.2)	13 (100)	
	Total	3 (7.7)	13 (33.3)	23 (59)	39 (100)	
12^th^ session	Control	1 (7.7)	2 (15.4)	10 (76.9)	13 (100)	0.230
	810 nm laser	2 (15.4)	2 (15.4)	9 (69.2)	13 (100)	
	940 nm laser	0 (0.0)	3 (23.1)	10 (76.9)	13 (100)	
	Total	3 (7.7)	7 (17.9)	29 (74.4)	39 (100)	

In this table showed the frequency and percentage of thermal test detection. The detection frequency of this test was the variant between the 3 groups. The ability of thermal detection had been improved in all groups until the 3rd session and then remained unchanged until the end of the 12th session. After 3 treatment sessions, almost all patients were able to detect the thermal stimuli and there was no significant difference between the 3 groups regarding non-detection and any detection of the thermal stimuli.

The intensity of dysesthesia and satisfaction were evaluated with a numerical scale: 0 meant entirely dissatisfied, and 10 meant completely satisfied in both control and examined groups. CNT was evaluated before and after treatment sessions (after the 1^st^, 3^rd^, 7^th^, and 12^th^ sessions).

### Statistical analysis

Data were analyzed with SPSS 26 using repeated measures ANOVA, Chi-square test, and Tukey tests at <0.05 level of significance. In addition, data distribution was evaluated using Kolmogorov-Smirnov test. 

## Results

This single-blinded randomized clinical trial studied 39 patients, including 21 women (53.8%) and 18 men (46.2%). Based on the Chi-squared test, there was no significant gender disparity between the three groups (*p*= 0.73). The mean and standard deviation (SD) of dysesthesia, 2-point discrimination, and patient satisfaction are shown in Table 3.

**Table 3:** Mean ± SD of dysesthesia, 2-point discrimination, and patients’ satisfaction

Variables	Groups (n=13)	Before treatment	1^st^ Session	3^rd^ Session	7^th^ Session	12^th^ Session
Dysesthesia	Control	8.00±1.08	6.53±1.19	5.15±1.51	4.30±1.65	4.15±1.21
	810 nm laser	8.00±1.29	5.00±1.22	3.53±1.12	2.53±0.87	2.46±1.126
	940 nm laser	7.846±1.28	6.38±0.86	4.53±1.198	3.23±1.42	2.23±1.48
Two Point Discrimination	Control	20.31±5.23	18.23±4.64	16.15±4.00	15.62±3.71	14.85±4.08
	810 nm laser	20.46±5.62	16.92±5.25	15.46±5.30	13.62±4.48	12.54±4.12
	940 nm laser	21.38±6.92	19.00±5.58	16.62±6.23	15.77±5.92	13.62±3.75
Patient’s satisfaction	Control	-	5.08±1.04	7.00±1.53	7.00±2.00	6.92±1.93
	810 nm laser	-	7.23±1.36	8.15±1.21	8.62±1.19	8.85±0.80
	940 nm laser	-	4.54±0.78	6.15±1.57	7.46±1.27	8.15±1.46

In this table shows the mean ± SD of dysesthesia, 2-point discrimination, and patients’ satisfaction in different sessions. Patients treated with lasers showed less dysesthesia in all sessions. The performance of patients was the variant in different sessions regarding 2-point discrimination. Patients represented more satisfaction after using the 810nm laser in all sessions but using the 940nm laser led to the least satisfaction amongst patients until the 7th session in which the satisfaction showed growth and overtook the control group.

Regarding dysesthesia, a repeated measures ANOVA showed a statistically significant interaction effect between the groups and time points, Wilks’ lambda= .473, F (8, 66) =3.747, *p*= 0.001. In addition, the repeated measures ANOVA with a Huynhfeldt correction showed that mean dysesthesia differed significantly between time points [F(3.602, 129.675)= 154.733, *p*< 0.001]. Pairwise comparisons between the three groups using the Bonferroni correction showed a significant difference between the 810nm laser and the control group (*p*= 0.001). Using the Tukey test showed the mean dysesthesia of the 810nm laser group was significantly lower than the control group in all treatment sessions: 1^st^ (*p*= 0.003), 3^rd^ (*p*= 0.008), 7^th^ (*p*= 0.006), and 12^th^ (*p*=0.005).

However, the pairwise comparisons showed no considerable differences between either the 810nm laser and the 980nm laser (*p*= 0.347) or the 980nm laser and the control group (*p*= 0.073). The pairwise comparisons between treatment sessions also revealed no statistically significant difference in dysesthesia between sessions 7^th^ and 12^th^ (*p*= 0.339).

In terms of 2-point discrimination, the repeated measures ANOVA showed no significant interaction effect between the groups and time points, Wilks’ lambda= .800, F(8,66)=.974, *p*= 0.464. The results also revealed that the main effect of the treatment group on the mean 2-point discrimination across time was not considerable (F (2, 36) =.403, *p*= 0.671). However, the main effect of time on this measure was statistically significant (Wilks’ lambda= .312, F (4, 33) =18.197, *p*< 0.001). 

The patient satisfaction analysis showed a statistically significant interaction effect between the groups and time points based on the repeated measures ANOVA (Wilks’ lambda= .643, F (6, 68) =2.805, *p*= 0.017). This test showed that mean patient satisfaction differed significantly between time points (sphericity assumed F (3, 108) = 35.754, *p*< 0.001). Pairwise comparisons between the three different groups using the Bonferroni correction showed a significant difference between the 810nm laser and both the control group (*p*< 0.001) and the 940nm laser (*p*< 0.001). Based on the Tukey test, the specimens of the 810nm laser group showed higher satisfaction than the control group after the 1^st^ (*p*< 0.001), 7^th^ (*p*= 0.028), and 12^th^ sessions (*p*= 0.006). Also this satisfaction was significantly higher than the 940nm laser group in the 1^st^ (*p*< 0.001) and 3^rd^ sessions (*p*= 0.003).

However, no significant difference was found between the 940nm laser and the control group (*p*= 1.00) in the pairwise comparisons. The treatment sessions’ pairwise comparison showed no significant difference between the 3^rd^, 7^th^, and 12^th^ sessions (*p*= 0.066 and *p*= 1.00, respectively). 

Based on the Chi-square test, there was no significant difference between the three groups regarding pinprick nociception (Table 4), brushstroke (Table 1), and thermal discrimination (Table 2).

**Table 4:** The frequency and the percentage of pinprick detection

Evaluation session	Groups	Detected N.(%)	Non-detected N.(%)	Total N.(%)	*p* Value
Before treatment	Control	7 (53.8)	6 (46.2)	13 (100)	0.899
	810 nm laser	8 (61.5)	5 (38.5)	13 (100)	
	940 nm laser	8 (61.5)	5 (38.5)	13 (100)	
	Total	23 (59)	16 (41)	39 (100)	
1^st^ session	Control	7 (46.2)	6 (53.8)	13 (100)	0.482
	810 nm laser	9 (69.2)	4 (30.8)	13 (100)	
	940 nm laser	7 (53.8)	6 (46.2)	13 (100)	
	Total	22 (56.4)	17 (43.6)	39 (100)	
3^rd^ session	Control	7 (46.2)	6 (53.8)	13 (100)	0.901
	810 nm laser	5 (38.5)	8 (61.553.8)	13 (100)	
	940 nm laser	6 (46.2)	7 (53.8)	13 (100)	
	Total	17 (43.6)	22 (56.4)	39 (100)	
7^th^ session	Control	4 (30.8)	9 (69.2)	13 (100)	0.892
	810 nm laser	4 (30.8)	9 (69.2)	13 (100)	
	940 nm laser	5 (38.5)	8 (61.5)	13 (100)	
	Total	13 (33.3)	26 (66.7)	39 (100)	
12^th^ session	Control	8 (61.5)	5 (38.5)	13 (100)	0.064
	810 nm laser	3 (23.1)	10 (76.9)	13 (100)	
	940 nm laser	3 (23.1)	10 (76.9)	13 (100)	
	Total	13 (35.9)	26 (64.1)	39 (100)	

In this table shows the frequency and percentage of Pin Prick detection. In almost all sessions, patients treated with both types of lasers did not show less detection of Pin Prick in comparison to the control group. Only after the first session, patients in 810nm laser could detect Pin Pricks more than the other 2 groups

## Discussion

Various treatments have been performed with lasers since 1970 in medicine and dentistry. There has been less reported postoperative pain or bleeding associated with the use of lasers in surgery sites, and it makes this approach as reliable, safe, and efficient treatment procedure [ 24 - 25 ]. For healing purposes, there are many photobiomodulation (PBM) protocols using wavelengths, energy density, and power density [ 26 - 30 ]. An in-depth and comprehensive description of PBM's uses in neurorehabilitation has not been found until now [ 30 - 31 ]. This study aims to compare the effect of two types of diode lasers: 940nm and 810nm, on the repair of inferior alveolar sensory nerve injuries.

In this study, the 810nm laser showed a significantly better performance in reducing dysesthesia than the control group in all 4 sessions (1^st^, 3^rd^, 7^th^, and 12^th^). The results of our study also revealed that both 810nm and 940nm lasers have similar effects on inferior alveolar nerve regarding factors, namely 2-point discrimination, brushstroke, thermal discrimination, and pinprick nociception; this similarity might be because of our short treatment period, which was one month. However, the healing caused due to PBM can be seen from 2 weeks to 1 year after irradiation [ 18 ].

Lasers with wavelengths between 808 and 830 nm are the most commonly used lasers in the field of neurorehabilitation [ 10 , 31 - 35 ], and the 810nm diode laser stands out among the rest owing to its considerable effect in previous studies which may arise the hypothesis that this laser might be the golden standard in low-level laser therapy (LLLT) in the near future [ 10 , 21 , 31 , 36 ]. In a recent systematic review, the effectiveness of LLLT was found considerable after more than one month [ 36 ]. In Mohajerani *et al*.’s study [ 21 ], as well; positive effects of 810nm laser on inferior alveolar nerve healing based on 2-point discrimination and brushstroke examination were revealed after six months. In two studies conducted by Hakimiha *et al*. [ 10 , 31 ], an improvement has been observed in neurosensory status after receiving PBM treatment by means of an 810nm laser. This laser can also improve the performance of the injured inferior alveolar nerve due to sagittal split osteotomy (SSO) when used with a light-emitting diode (LED) [ 21 ]. In addition, 810nm lasers can improve pain threshold and treat crushed inferior alveolar nerves based on previous studies [ 1 , 10 ].

The biological mechanisms of low-level lasers’ effects on nerves have not been precisely found. A recent hypothesis declares that infrared irradiation (790 to 830nm wavelength) can enhance the production of adenosine triphosphate (ATP) and proteins in nerve fibers. A terminal enzyme of the electron transport chain, cytochrome c oxidase (CCO), absorbs the light. These enzymes oxidize oxygen for energy metabolism, accelerating electron transfer reactions via photodissociation of inhibitory nitric oxide (NO). The non-covalent bond of NO to CCO's heme and copper centers blocks oxygen competitively at a ratio of 1:10, resulting in an increase in mitochondrial membrane potential (MMP) and ATP, cyclic adenosine monophosphate (cAMP), and reactive oxygen species levels [ 37 ]. 

This wavelength of light can increase intracellular calcium and, because of the stimulation of rhodopsin kinase and photosensitive fibers, can affect healing processes [ 33 - 34 , 38 - 39 ]. Calcium enters the cell through light-sensitive ion channels. When the first photon is absorbed, the oxygen radical, cAMP, nitric oxide, and calcium ions produce, which results in transcription activation. There are several physiological effects associated with all of these events, including an increase in genes pertaining to protein synthesis, cell migration, proliferation, anti-inflammatory signaling, and anti-apoptotic proteins [ 37 ].

LLLT has excellent outcomes regarding nerve healing. It is an effective method for healing different inferior alveolar nerve injuries [ 21 , 23 ]. In the present study, all patients with at most six months passed from their nerve injury were studied, which might be important in the nerve healing process. Some previous studies found that receiving PBM sooner than six months after inferior alveolar nerve damage can lead to better results, like a higher visual analogue scale (VAS) test recovery rate than receiving it later [ 1 , 18 ]. Using PBM seems more comfortable for patients rather than surgical or pharmacological treatment plans, as the results of this study showed the patients’ satisfaction was higher in the 810nm laser group than the control one receiving no laser irradiation. The unpredictable recovery outcomes from nerve injury can result in avoiding surgery-related protocols for patients with sensory defects [ 1 , 8 , 22 , 32 , 38 , 40 ].

Despite the strengths of this study, there were some limitations as well. This study was single blind and the patients were aware of the treatment procedure. Since the control group received only medications with no laser irradiation, it might lead to a placebo effect. So, further double-blind studies are suggested. PBM is reported to have fewer side effects than pharmacotherapy [ 22 ]. In this study, we did not evaluate the effects of LLLT versus pharmacotherapy, and we cannot determine the proportion of medication effects versus laser therapy. In addition, due to the special conditions of the participants, it was not possible to increase the number of treatment sessions or reduce the distance between the sessions to a larger extent. Therefore, we are not able to evaluate the side effects of this approach in the long term which can be analyzed in future studies.

## Conclusion

Our study showed that 810nm diode laser could reduce dysesthesia and enhance patient satisfaction more than a 940nm diode laser. However, there were no significant differences between the three groups regarding clinical neurosensory tests. As there is no exact protocol for PBM, more studies need to be conducted to clarify the effects and side effects of this approach.
